# Impact of COVID-19 Pandemic on Disease Control Status and Quality of Life of Patients with Acromegaly

**DOI:** 10.3390/medicina58121711

**Published:** 2022-11-23

**Authors:** Rok Herman, Andrej Janež, Katja Goričar, Manfredi Rizzo, Mojca Jensterle

**Affiliations:** 1 Department of Endocrinology, Diabetes and Metabolic Diseases, University Medical Center Ljubljana, 1000 Ljubljana, Slovenia; 2 Department of Internal Medicine, Faculty of Medicine, University of Ljubljana, 1000 Ljubljana, Slovenia; 3 Pharmacogenetics Laboratory, Institute of Biochemistry and Molecular Genetics, Faculty of Medicine, University of Ljubljana, 1000 Ljubljana, Slovenia; 4 Department of Health Promotion, Mother and Child Care, Internal Medicine and Medical Specialties (Promise), University of Palermo, 90127 Palermo, Italy

**Keywords:** acromegaly, disease control status, SAGIT instrument, AcroQoL, COVID-19 pandemic, public health measures

## Abstract

*Background and Objectives*: Despite the best efforts of healthcare workers and the deployment of alternative healthcare delivery solutions through telemedicine, the pandemic has disrupted standard care for patients with chronic conditions. The long-lasting pandemic has also had a profound impact on the quality of life (QoL) of the majority of patients with chronic illnesses. The management of rare diseases has been particularly challenging. We aimed to evaluate the impacts that the long-lasting pandemic had on the disease control status and QoL in patients with acromegaly. *Materials and Methods*: Our prospective study included 34 patients from a national referral centre. The baseline SAGIT and AcroQoL results were obtained in October 2020 during the lockdown period of the SARS-CoV2 pandemic. The follow-up results were assessed during the summer of 2022 in a period without any public health restrictions. All the patients were additionally evaluated for their attitude towards preventative public health measures against SARS-CoV2 spread and required mask wearing during the pandemic. *Results*: By comparing assessments in 2020 during the lockdown period and 2022 post-lockdown, we observed some improvement in SAGIT subscores T and I, most likely reflecting treatment changes in a small number of patients. The global SAGIT score remained stable. QoL measurement by AcroQoL did not demonstrate any changes. There was a negative correlation between SAGIT subscore S and the AcroQoL results. We also noted that the group of patients with the most negative attitude toward public health measurements for preventing SARS-CoV2 spread had higher AcroQoL results than others. *Conclusion*: Our results showcase that the SARS-CoV2 pandemic, lasting over two years, did not impact the disease control status and QoL in patients with acromegaly. The cohort continued to be well controlled and without changes in QoL. We measured a relatively favourable attitude towards the public health measures to prevent the spread of SARS-CoV2; in particular, patients who had a lower QoL had more positive attitudes towards these measures.

## 1. Introduction

Improvement in acromegaly treatment options enables the achievement of biochemical control and normal life expectancy in the vast majority of patients [1]. However, patients with acromegaly often face a poor quality of life (QoL) that is independent of biochemical disease control [1,2]. The frequently observed discordance between patient and clinician perception of the disease control status could be bridged using the new patient- and clinician-reported outcome tools [3]. The clinician-reported outcome tools, such as the SAGIT instrument (SAGIT) and acromegaly disease activity tool (ACRODAT), provide an objective measure of present signs and symptoms, comorbidities, tumour profile, and biochemical parameters [4,5]. On the other hand, the patient-assessed acromegaly symptom questionnaire (PASQ), the acromegaly quality of life questionnaire (AcroQoL), the acromegaly treatment satisfaction questionnaire (AcroTSQ), the enlargement of the extremities questionnaire, and the acromegaly comorbidities and complaints questionnaire allow patients to give their perspectives on current symptoms and QoL [6,7].

The SARS-CoV2 pandemic represents the toughest challenge faced by healthcare systems in modern history through its effect on altered resource allocation, the demand for innovative solutions for patients’ care, and unavoidable deviations in the quality and continuum of care for chronic and rare diseases. Despite the best efforts of healthcare workers and the deployment of alternative healthcare delivery solutions through telemedicine, the pandemic has disrupted standard care for patients with chronic conditions, and its effect on disease control status is currently explored in multiple conditions, including pituitary-related diseases [8,9,10,11,12,13,14,15,16].

Lasting over two years, the pandemic has also profoundly impacted the mental well-being and QoL of the majority of patients with chronic and acute illnesses [17,18,19,20]. In addition to direct health impact, it has numerous indirect effects on people with chronic diseases through various restrictions, lockdowns, separation from family and friends, and economic hardships [8,21,22].

The direct impact of the SARS-CoV2 pandemic on QoL in patients with acromegaly has been evaluated in only one study to date that demonstrated stable AcroQoL results when comparing the assessment two years before the pandemic and during the first lockdown period in 2020 [9]. The aim of our study was to provide an objective evaluation of the impacts that the SARS-CoV2 pandemic had on the disease control status and the QoL. We also investigated the associations between the patients’ attitudes towards preventative public health measures during the pandemic and their QoL.

## 2. Patients and Methods

### 2.1. Design

Our prospective study included 34 patients from a national referral centre who had previously participated in a cross-sectional study of the clinical applicability of patient- and clinician-reported outcome tools in the management of patients with acromegaly [23], and decided to participate in the long-term follow-up. There were no inclusion restrictions on age, sex, treatment, or disease control status. Given the observational nature of the study, the management and therapeutic approaches of the different physicians were chosen according to their expertise, with no influence from this study.

The baseline SAGIT and AcroQoL results were assessed in October 2020 during the lockdown period of the SARS-CoV2 pandemic, when a significant degree of in-person appointments was replaced with telemedicine and strict public health measures focusing on personal hygiene and social distancing campaigns still strictly in place. The follow-up SAGIT and AcroQoL results were assessed during the summer of 2022, with normally functioning in-person appointments, the dominance of milder SARS-CoV2 variants, high vaccination or previous infection rates in the population, and, in general, a more favourable COVID-19 disease course, with almost no public health restrictions to limit the spread of SARS-CoV2 in place.

The study was approved by the national medical ethics committee, and conducted according to the guidelines of the Declaration of Helsinki and its later amendment. All the subjects provided informed written consent to participate in a study and to have their data published in a journal article.

### 2.2. Patients

All the included patients were diagnosed and treated between the years 2000 and 2022, and regularly followed up at our centre. An initial diagnosis of acromegaly was confirmed by the IGF-1 levels above the gender- and age-adjusted upper limit of normal (ULN), lack of suppression of GH levels to <0.4 μg/L during a 75-g oral glucose tolerance test (OGTT), or random GH levels > 1 μg/L (patients with diabetes) and a pituitary adenoma visualized with magnetic resonance imaging (MRI). From the pool of 72 patients included in the baseline assessment during the initial lockdown period of the SARS-CoV2 pandemic, 41 responded to the invitation for follow up. In the end, 34 patients met the inclusion criteria of having filled the AcroQoL questionnaire correctly at both assessments and having available enough data to accurately determine the SAGIT score.

Patients were classified as either cured after surgery, controlled on pharmacotherapy, or uncontrolled. Acromegaly was considered cured after transsphenoidal surgery if the nadir GH level during an OGTT was <0.4 μg/L, and age- and gender-adjusted IGF-1 levels normalized. Patients were classified as controlled on pharmacotherapy if age- and gender-adjusted IGF-1 levels normalized.

Serum GH concentrations were measured using the Immulite2000 analyser and chemiluminescent immunoassay (CLIA) kits »Immulite 2000 Growth Hormone (hGH, Recombinant 98/574)« (both Siemens Healthcare Diagnostics Products Ltd., Camberley, UK). Serum IGF-I concentrations were measured using an iSYS analyser and chemiluminescent immunoassay (CLIA) kits »IDS-iSYS Insulin-like Growth Factor-I (IGF-I)« (both Immunodiagnostic Systems Limited, East Boldon, UK).

### 2.3. Patient- and Clinician-Reported Outcome Tools

The SAGIT has been developed to enable accurate characterisation of acromegaly disease activity and comprises five subscores: signs and symptoms (Score S: 0–4), associated comorbidities (Score A: 0–6), GH level (Score G: 0–4), IGF-1 level (Score I: 0–3), and tumour profile (Score T: 0–5) [5]. The scoring was conducted using the version of SAGIT used in Step 2 of the pilot study [5].

The AcroQoL developed by Webb et al. [24] was used for the QoL measurement, and was already linguistically adapted and validated in the Slovenian language. The questionnaire is divided into a physical scale (8 items) and a psychological scale (14 items). The latter is further subdivided into additional subscales: appearance (7 items) and personal relations (7 items). The maximum score is 110 points, while the worst score is 22. The resulting scores were standardized on a scale running from 0 (worst QoL) to 100 (best QoL) by using a formula described in the literature [24]. At both measurements, all the participants had their AcroQoL score determined.

All the patients were additionally asked two questions regarding their attitude towards preventative public health measures against SARS-CoV2 spread (Question 1) and the required mask wearing during the pandemic (Question 2). Their responses were therefore classified from 1 to 3, with one corresponding with the most favourable attitude and three with the most critical viewpoint.

Question 1: »Which of the following options best describes your general attitude towards all the preventative health measures that were in place over the last two years in order to limit the SARS-CoV2 spread?

1. In general, I believe that the public health and social measures during the last two years were not strict enough.
2. In general, I believe that the public health and social measures during the last two years were justified and suitable.
3. In general, I believe that the public health and social measures during the last two years were unnecessary and too harsh.«

Question 2: »Which of the following options best describes your attitude towards the required mask-wearing over the last two years in order to limit the SARS-CoV2 spread?

1. I find the required mask-wearing to be pleasurable.
2. The requirement to wear masks in public places does not bother me.
3. I am against the required mask-wearing in public spaces.«

### 2.4. Statistics

Non-normally distributed continuous data are expressed as medians with interquartile ranges (25–75%). We used Spearman’s rho coefficient (ρ) for calculating the correlations between continuous study variables. A Wilcoxon signed–rank test was used to compare continuous study variables for related samples (comparison of different time points). The non-parametric Mann–Whitney test was used to compare continuous study variables between different groups. A *p* value below 0.05 was considered statistically significant. All the statistical analyses were performed using IBM SPSS Statistics, version 27.0 (IBM Corporation, Armonk, NY, USA).

## 3. Results

### 3.1. Patients and Clinically Evaluated Acromegaly Control Status

Our cohort included 24 women (70.6%) and 10 men (29.4%), with a median age of 59.5 (50–69) years. At the baseline measurement, 26 patients (76.5%) were classified as cured or controlled on pharmacotherapy, and 8 (23.5%) patients as uncontrolled. At the follow up, 31 patients (91.2%) were cured or controlled, and 3 (8.8%) were uncontrolled. In 2020, 17 patients were receiving pharmacotherapy (11 monotherapy and 6 combination therapy) compared to 16 patients in 2022 (9 monotherapy and 7 combination therapy). Between both assessments, two patients were operated on, and seven patients had their pharmacotherapy increased either by increasing the dosage of monotherapy or switching from monotherapy to combination therapy. None of the patients underwent treatment with radiotherapy. More detailed patients’ characteristics at the time of the diagnosis, baseline assessment, and follow-up measurement are presented in Table 1.

### 3.2. Changes in SAGIT and AcroQoL Results over Time

Table 2 compares SAGIT scores and AcroQoL results between the baseline and follow-up assessments corresponding to the disease control over time. It also specifies the number of patients with increased or reduced scores for any category. With SAGIT, we noticed a significant improvement in subscores I and T (*p* = 0.047 and *p* = 0.006, respectively), even though both absolute scores remained unchanged in 25 and 23 patients, respectively. Other SAGIT subscores, as well as the global SAGIT score, remained unchanged. With AcroQoL, we noticed increased results for all categories except the physical scale, with the global AcroQoL score rising from 62.5 (50–82.1) to 73.9 (54.3–86.6); however, none of the differences were statistically significant (all *p* > 0.05).

### 3.3. Correlations between SAGIT and AcroQoL Results

Table 3 shows the correlations between the subscores from the SAGIT and AcroQoL for the follow-up assessment. The significant correlations for the baseline were already reported [23]. We observed many statistically significant negative correlations between subscore S, and the global SAGIT score and AcroQoL results. The global SAGIT and AcroQoL scores had a Spearman’s ρ value of −0.383, *p* = 0.025. We also explored the correlations between the changes (follow up in 2022 vs. baseline in 2020) in the global SAGIT score and the changes in all the AcroQoL results. Despite some correlations having a positive value, no significant associations were observed. For the change in the global AcroQoL score, Spearman’s ρ was 0.219 (*p* = 0.228).

### 3.4. Attitude towards Pandemic Public and Social Health Measurements and AcroQoL

#### 3.4.1. General Attitude

For the first question regarding the general attitude towards the pandemic public health measures, 3 patients (8.8%) answered with the first option (»In general, I believe that public health and social measures during the last two years were not strict enough«), 25 patients (73.5%) chose the second answer (»In general, I believe that public health and social measures during the last two years were justified and suitable«), and 6 patients (17.6%) opted for the last answer (»In general, I believe that public health and social measures during the last two years were unnecessary and too harsh«). The median results for all the AcroQoL scales increased from the first to the last answer (most favourable to least favourable attitude). Due to the low number of patients that chose the first answer and a similar acceptable attitude toward preventative measures, we combined patients that selected the first and the second answer into one category with 28 patients (82.3%). Table 4 presents the difference in AcroQoL results between the groups (Answer 1 + 2 vs. Answer 3). In all the categories except Appearance, the group with the most unfavourable attitude toward the pandemic measures (Answer 3) had significantly higher scores, with a global score of 82.4 (76.7–92.3) compared to 64.2 (53.4–84.9) in the other group (Answer 1 + 2), *p* = 0.037.

#### 3.4.2. Mask Wearing

For the second question regarding the attitude towards the required mask wearing in public places, 1 patient (3.0%) answered with the first option (»I find the required mask wearing to be pleasurable.«), 28 patients (82.4%) with the second option (»The requirement to wear masks in public places does not bother me.«), and 5 patients (15.2%) opted for the last answer (»I am against the required mask wearing in public spaces.«). Due to the low number of patients that chose the first answer and a similar positive attitude towards mask wearing, we combined the first and the second answer into one category that had 29 patients (85.4%) for the comparison of the AcroQoL results between different groups (Answer 1 + 2 vs. Answer 3). The AcroQoL scores did not differ between both groups (Table 5).

## 4. Discussion

By comparing the baseline assessment during the lockdown period of the SARS-CoV2 pandemic in 2020 and follow up during the post-lockdown period in the summer of 2022, we observed improvement in SAGIT subscores T and I, most likely reflecting treatment changes in a small number of patients. However, the disease control status assessed by the SAGIT global score remained stable. Similarly, QoL measurement by AcroQoL did not demonstrate any changes. In addition, we observed significant negative correlations between SAGIT subscore S, and the global SAGIT score and AcroQoL results. Lastly, we noted that the group of patients with the most negative attitude toward public health measurements for preventing SARS-CoV2 spread had higher AcroQoL results than others.

Based on our results, we can conclude that the alternative healthcare delivery methods during the pandemic did not affect the patients’ perception or objective markers of acromegaly control. The chronic care of our patients remained at a high level throughout the pandemic. Similar conclusions were observed in the Northwest Italy Regional hub for endocrine diseases by Gatto et al. Despite the initial 33% reduction of on-site evaluations during the first lockdowns, that reduction was partially mitigated by performing telephonic follow ups, and the percentage of controlled patients did not change significantly [15]. Furthermore, the pandemic and its consequent limitations did not seem to have affected outpatient access and the achievement of good disease control in a sample of 41 acromegalic patients with SAGIT, AcroQoL, and ACRODAT results comparable between 2018 and 2020–2021 assessments [9]. A different perspective can be gained from the results of the questionnaire drafted by a steering committee of acromegaly experts, including answers from 84 endocrinologists in which just 21.4% of respondents reported no negative effects from the pandemic on diagnostic practice patterns, and just 19.1% reported no negative impact on patient follow-up practices [11]. Interestingly, many respondents (55.9%) indicated that remote methods had improved their ability to communicate with their patients, and 69.0% indicated that they would continue to use methods of consultation necessitated by the pandemic [11]. The positive experience with the new healthcare delivery methods for acromegaly patients during the pandemic through virtual meetings, simplification of the blood sample collection, and the digitalization of prescriptions was also described in Brazil [25]. Combined, experiences throughout the pandemic could be a building stone for the creation of a novel continuum of care for patients with acromegaly that will be better suited to their individual needs.

Moreover, in our cohort, the AcroQoL result insignificantly increased from the first to the last measurement, and the median global score of 73.9 continues to be relatively high compared to previously published study cohorts [26,27,28]. The scale with the best result was the personal relations subscale, while the most impaired was the physical scale. Concerns for self-health due to COVID-19 have already been associated with a positive attitude toward health protection measures [29,30]. In our cohort, 82.3% of patients thought that all the protective measures over the last two years were suitable or even not strict enough, which is substantially higher than what was reported from general population screening in Slovenia. Based on a survey of 628 people at the beginning of 2022, 37.6% of participants from the general population thought the measures were too harsh and unjustified; 53.2% that the measures were suitable and justified; and 9.1% perceived them as not strict enough [31]. In combination with the result that patients who saw public health and social measures favourably generally had a lower QoL, it is feasible that patients who perceive their acromegaly status as worse also perceive a bigger threat in this pandemic and that, in general, acromegalic patients are more inclined to follow and see the rules favourably due to their chronic disease. However, it is impossible to confirm that hypothesis without a controlled study demonstrating the difference in the attitude between patients and healthy controls in a similar setting because the fact that patients were asked that question by someone perceived as a health authority might influence their answers.

We demonstrated negative correlations between SAGIT and AcroQoL. Larger cohorts are needed to confirm our findings, and simultaneously contrast them to the potential correlations between the biochemical control and QoL. The holistic approach of combining patients’ and clinicians’ perspectives will help to identify modifiable signs, symptoms, and comorbidities as individual treatment targets that might help clinicians improve QoL in this population.

## 5. Conclusions

In conclusion, our results showcase that the over two-years lasting SARS-CoV2 pandemic did not impact the disease control status and QoL in patients with acromegaly. The study cohort continued to be well controlled and without changes in QoL. The results may decrease our concerns when we may not be able to deliver health services in person. Furthermore, we measured a relatively favourable attitude towards the public health measures to prevent the spread of SARS-CoV2; particularly, the patients who had a lower QoL had more positive attitudes towards these measures. This has a particular importance during this long pandemic time, since patients with chronic conditions have been seriously exposed to a deterioration of their diseases and a higher prevalence of complications [32,33]; this is why we have proposed a comprehensive and multidisclipinary syndemic approach for patients with cardio-endo-metabolic disorders during the COVID-19 era [34,35,36]. Overall, our data emphasise the complementary nature of clinician- and patient-reported outcome tools in the management and follow up of acromegaly.

## Figures and Tables

**Table 1:** Patients’ characteristics at the time of diagnosis, at baseline of the study and at the follow up.

Presentation at Diagnosis N (%) or Median (25–75%)		
Age (years)	48.0 (41.0–57.5)	
Estimated diagnostic delay (years)	4.0 (2.0–11.0)	
Adenoma classification	Microadenoma	Macroadenoma
	7 (21.9%)	25 (78.1%)
IGF-1 levels (ng/mL)	709 (493.9–837.1)	
Characteristics at baseline N (%) or median (25–75%)		
IGF-1 levels (ng/mL)	162 (133–191)	
Patients on pharmacological therapy	17 (50.0)	
Type of pharmacological therapy	Somatostatin analogs (SRL)	8 (47.1)
	Dopamine agonists (DA)	2 (11.8)
	Pegvisomant	1 (5.9)
	SRL + pegvisomant	6 (35.3)
	SRL + DA	0 (0.0)
Characteristics at follow up N (%) or median (25–75%)		
IGF-1 levels (ng/mL)	137 (114.5–164)	
Patients on pharmacological therapy	16 (47.1)	
Type of pharmacological therapy	Somatostatin analogs (SRL)	7 (43.8)
	Dopamine agonists (DA)	1 (6.3)
	Pegvisomant	1 (6.3)
	SRL + pegvisomant	6 (37.5)
	SRL + DA	1 (6.3)

**Table 2:** The comparison of SAGIT scores and AcroQoL results between both assessments.

	Baseline Median (25–75%)	Follow Up Median (25–75%)	*p*	Change N (%)
**SAGIT**				
**Subscore S**	1 (1–2)	1 (0.8–2)	0.917	⇑ 5
				≈ 22
				⇓ 5
**Subscore A**	2 (1–2)	2 (1–2)	0.077	⇑ 12
				≈ 16
				⇓ 4
**SubScore G**	0 (0–0)	0 (0–0)	0.414	⇑ 1
				≈ 29
				⇓ 2
**Subscore I**	0 (0–0)	0 (0–0)	**0.047**	⇑ 1
				≈ 25
				⇓ 6
**Subscore T**	0 (0–1)	0 (0–0)	**0.006**	⇑ 0
				≈ 23
				⇓ 9
**Global SAGIT Score**	4 (2.3–5)	3 (3–4)	0.433	⇑ 10
				≈ 6
				⇓ 16
**AcroQoL**				
**Physical Scale**	65.6 (43.8–78.1)	65.6 (50–82.0)	0.545	⇑ 15
				≈ 6
				⇓ 13
**Psychological Scale**	72.3 (50.9–86.2)	78.6 (56.7–89.3)	0.097	⇑ 17
				≈ 3
				⇓ 14
**Appearance**	66.1 (45.5–86.6)	71.4 (48.2–85.7)	0.125	⇑ 16
				≈ 5
				⇓ 13
**Personal Relations**	78.6 (60.7–89.3)	82.1 (63.4–96.4)	0.091	⇑ 18
				≈ 5
				⇓ 11
**Global AcroQoL Score**	62.5 (50–82.1)	73.9 (54.3–86.6)	0.213	⇑ 16
				≈ 4
				⇓ 14

⇑ increase in the score; ≈ the score remained the same; ⇓ decrease in the score.

**Table 3:** Correlations between SAGIT and AcroQoL scores at follow up.

		Physical	Psychological	Appearance	Personal Relations	Global Score
**Subscore S**	ρ	−0.421	−0.373	−0.255	−0.422	−0.387
	*p*	**0.013**	**0.030**	0.145	**0.013**	**0.024**
**Subscore A**	ρ	−0.300	−0.254	−0.201	−0.287	−0.273
	*p*	0.085	0.148	0.253	0.100	0.118
**Subscore G**	ρ	0.070	0.126	0.192	0.065	0.079
	*p*	0.694	0.478	0.278	0.713	0.656
**Subscore I**	ρ	0.023	0.156	0.060	0.233	0.102
	*p*	0.897	0.378	0.734	0.184	0.565
**Subscore T**	ρ	−0.254	−0.274	−0.239	−0.280	−0.284
	*p*	0.147	0.117	0.174	0.109	0.104
**Global SAGIT Score**	ρ	−0.455	−0.337	−0.238	−0.390	−0.383
	*p*	**0.007**	0.051	0.175	**0.023**	**0.025**

**Table 4:** The difference in AcroQoL results between different groups divided by general attitude towards preventative public health measures during the pandemic.

	Answer 1 + 2	Answer 3	*p*
**Physical Scale**	60.9 (45.3–75)	78.1 (68–90.6)	**0.042**
**Psychological Scale**	71.4 (54–89.3)	83.9 (80.4–96)	**0.037**
**Appearance**	67.9 (42.9–84.8)	76.8 (70.5–94.6)	0.133
**Personal Relations**	75 (58–92.9)	94.6 (89.3–100)	**0.017**
**Global AcroQoL Score**	64.2 (53.4–84.9)	82.4 (76.7–92.3)	**0.037**

**Table 5:** The difference in AcroQoL results between different groups divided by general attitude towards the required mask wearing during the pandemic.

	Answer 1 + 2	Answer 3	*p*
**Physical Scale**	65.6 (50–82.8)	68.8 (48.4–87.5)	0.637
**Psychological Scale**	75 (56.3–89.3)	80.4 (58.9–91.1)	0.671
**Appearance**	71.4 (46.4–85.7)	71.4 (51.8–91.1)	0.851
**Personal Relations**	78.6 (62.5–96.4)	92.9 (58.9–96.4)	0.637
**Global AcroQoL Score**	70.5 (54–86.9)	77.3 (55.1–89.2)	0.637

## Data Availability

The authors agree to make data and materials supporting the results or analyses presented in this paper available upon reasonable request.
